# Supplementary material for: Activation-inducible CAR expression enables precise control over engineered CAR T cell function
Source: Commun Biol. 2023 Jun 5;6:604. doi: 10.1038/s42003-023-04978-w (PMC10241805; doi:10.1038/s42003-023-04978-w)
Supplement: Supplementary file 2 — Supplementary Information [file 42003_2023_4978_MOESM2_ESM.pdf]

Activation-inducible CAR expression enables precise control over engineered CAR T cell function

Simon P. Fraessle[1#], Claudia Tschulik[1#], Manuel Effenberger[1#*], Vlad Cletiu[1], Maria Gerget[1], Kilian Schober[2], Dirk H. Busch[2], Lothar Germeroth[1], Christian Stemberger[1], Mateusz P. Poltorak[1]

Supplementary Figures

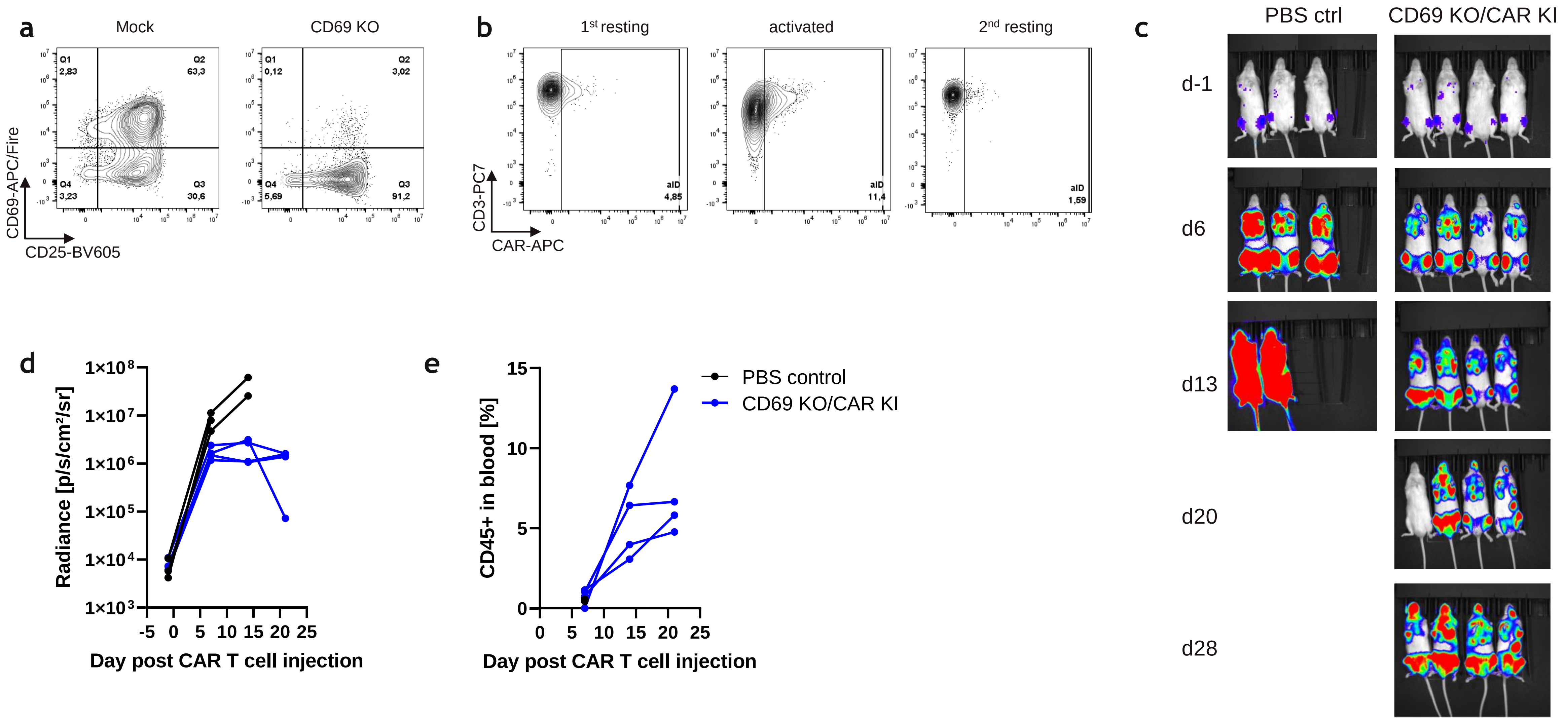

Supplementary Figure 1. CAR expression can also be driven by CD69 promotor. (a) Highly efficient CRISPR mediated CD69 KO in primary T cells. Physiological CD69 expression observed in mock control cells. CD69 KO and mock control sample was Expamer stimulated 24h before analysis to trigger CD69 upregulation. Cells are pre-gated on living, single CD3+ lymphocytes. **(b)** Activation inducible CAR T cells under control of CD69 promotor. CD69 engineered feedback loop CAR T cells were rested, polyclonally re-stimulated and subsequently rested a second time as depicted. Representative plots display increase in activation-induced CAR expression upon restimulation. Of note, CD3 expression is decreased in the restimulated sample which is indicative of T cell activation. (**c**) Tumor imaging of tumor bearing mice over time. Tumor was detected using IVIS bioluminescence measurements. Luminescence images from one representative experiment are shown. (**d**) Quantification of tumor images shown in (d) displaying PBS controls in black, CD69 KO/CAR KI cells in blue. 3-4 mice per group were analyzed. Each line represents corresponding mouse. (**e**) Transferred cells (CD45+) detected in the blood are shown, pre-gated on living lymphocytes. 3-4 mice per group were analyzed. Each line represents corresponding mouse.

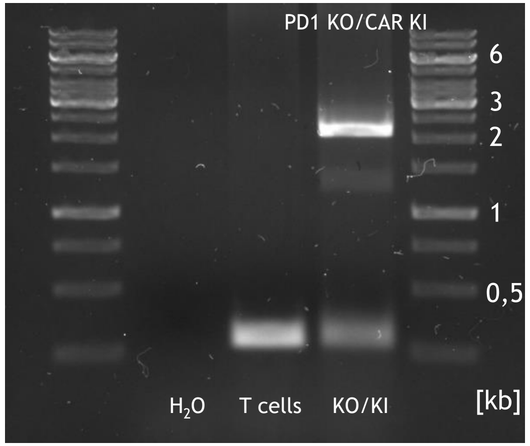

Supplementary Figure 2. Detection of CAR gene in genome. Gel indicates detection of CAR sequence under PD1 locus in edited and non-edited T cells via genomic DNA PCR.
