# Supplementary material for: Activation-inducible CAR expression enables precise control over engineered CAR T cell function
Source: Commun Biol. 2023 Jun 5;6:604. doi: 10.1038/s42003-023-04978-w (PMC10241805; doi:10.1038/s42003-023-04978-w)
Supplement: Supplementary file 5 — Reporting Summary [file 42003_2023_4978_MOESM5_ESM.pdf]

nature portfolio

Corresponding author(s): Dr. Manuel Effenberger

Last updated by author(s): May 19, 2023

Reporting Summary

Nature Portfolio wishes to improve the reproducibility of the work that we publish. This form provides structure for consistency and transparency in reporting. For further information on Nature Portfolio policies, see our Editorial Policies and the Editorial Policy Checklist.

Statistics

For all statistical analyses, confirm that the following items are present in the figure legend, table legend, main text, or Methods section.

n/a	Confirmed	
☐	☒	The exact sample size (*n*) for each experimental group/condition, given as a discrete number and unit of measurement
☐	☒	A statement on whether measurements were taken from distinct samples or whether the same sample was measured repeatedly
☐	☒	The statistical test(s) used AND whether they are one- or two-sided<br>*Only common tests should be described solely by name; describe more complex techniques in the Methods section.*
☒	☐	A description of all covariates tested
☒	☐	A description of any assumptions or corrections, such as tests of normality and adjustment for multiple comparisons
☐	☒	A full description of the statistical parameters including central tendency (e.g. means) or other basic estimates (e.g. regression coefficient) AND variation (e.g. standard deviation) or associated estimates of uncertainty (e.g. confidence intervals)
☐	☒	For null hypothesis testing, the test statistic (e.g. *F*, *t*, *r*) with confidence intervals, effect sizes, degrees of freedom and *P* value noted<br>*Give P values as exact values whenever suitable.*
☒	☐	For Bayesian analysis, information on the choice of priors and Markov chain Monte Carlo settings
☒	☐	For hierarchical and complex designs, identification of the appropriate level for tests and full reporting of outcomes
☒	☐	Estimates of effect sizes (e.g. Cohen's *d*, Pearson's *r*), indicating how they were calculated

Our web collection on statistics for biologists contains articles on many of the points above.

Software and code

Policy information about availability of computer code

Data collection	N/A
Data analysis	N/A

For manuscripts utilizing custom algorithms or software that are central to the research but not yet described in published literature, software must be made available to editors and reviewers. We strongly encourage code deposition in a community repository (e.g. GitHub). See the Nature Portfolio guidelines for submitting code & software for further information.

Data

Policy information about availability of data

All manuscripts must include a data availability statement. This statement should provide the following information, where applicable:

- Accession codes, unique identifiers, or web links for publicly available datasets
- A description of any restrictions on data availability
- For clinical datasets or third party data, please ensure that the statement adheres to our policy

The authors declare that data generated or analyzed for this study are available within the paper and its supplementary information (including Supplementary Data 1 file). Described findings are proprietary of Bristol-Myers Squibb Company and some information are considered a trade secret. The raw data that support the findings of this study are available from the authors but restrictions may apply to the availability of these data. Data are, however, available from the authors upon reasonable request and with permission from the Bristol-Myers Squibb Company.

Human research participants

Policy information about studies involving human research participants and Sex and Gender in Research.

Reporting on sex and gender	N/A
Population characteristics	N/A
Recruitment	N/A
Ethics oversight	Buffy coats were obtained from autologous adult female or male blood donors at the Institute for Anesthesiology, German heart center Munich (State of Bavaria and Technical University Munich). Written informed consent was obtained from each donor and usage of blood samples was approved according to national law by the local Institutional Review board and the declaration of Helsinki and Istanbul (Ethics committee of the faculty of Medicine, Technical University Munich: 360/13 and 55/14). Leukapheresis samples received from healthy donors were collected at the CCC Cellex Collection Center Dresden under the ethical quote (Ethical committee of the Technical University Dresden: EK309072016).

Note that full information on the approval of the study protocol must also be provided in the manuscript.

Field-specific reporting

Please select the one below that is the best fit for your research. If you are not sure, read the appropriate sections before making your selection.

☒ Life sciences ☐ Behavioural & social sciences ☐ Ecological, evolutionary & environmental sciences

For a reference copy of the document with all sections, see nature.com/documents/nr-reporting-summary-flat.pdf

Life sciences study design

All studies must disclose on these points even when the disclosure is negative.

Sample size	Sample size was selected base of standard experimental practices (mostly 3 or more independent experiments), unless number of repetition was technically challenging e.g., mouse studies. No statistical samples size calculations were performed.
Data exclusions	No data exclusion was done.
Replication	All data (except supplementary material) was replicated as least once as separate independent experiment.
Randomization	Allocation was random.
Blinding	Blinding was not relevant for this study. Samples (e.g, animals, blood donations) were random and the same operator was conducting experiment and analyzing the data in unbiased manner.

Reporting for specific materials, systems and methods

We require information from authors about some types of materials, experimental systems and methods used in many studies. Here, indicate whether each material, system or method listed is relevant to your study. If you are not sure if a list item applies to your research, read the appropriate section before selecting a response.

Materials & experimental systems

n/a	Involved in the study	
☐	☒	Antibodies
☐	☒	Eukaryotic cell lines
☒	☐	Palaeontology and archaeology
☐	☒	Animals and other organisms
☒	☐	Clinical data
☒	☐	Dual use research of concern

Methods

n/a	Involved in the study	
☒	☐	ChIP-seq
☐	☒	Flow cytometry
☒	☐	MRI-based neuroimaging

Antibodies

Antibodies used	CD3 (clone OKT3-PC7), CD4 (clone OKT4-BV421), CD8 (clone RPA-T8-BV510), CD45 (clone HI30), PD1 (clone EH12.2H7-FITC and -BV421), CD69 (clone FN50-APC/Fire), CD19 (clone HIB19-BV421 and -FITC), and CD25 (clone BC96-BV605). In addition, an anti-

	idiotype (CD19) antibody produced in-house coupled to APC (Bristol Myers Squibb) and Propidium Iodide (Thermo Fisher Scientific) was used.
Validation	Commercially available antibodies were used according to manufacturers' instructions and in-house antibodies were tested for specificity using positive and negative controls.

Eukaryotic cell lines

Policy information about cell lines and Sex and Gender in Research

Cell line source(s)	Commercially available B-cell-derived Lymphoblastoid cells (LCL), Human Embryonic Kidney cells (HEK293) and Raji cells were used
Authentication	N/A
Mycoplasma contamination	cell lines were tested negative for mycoplasma
Commonly misidentified lines (See ICLAC register)	N/A

Animals and other research organisms

Policy information about studies involving animals; ARRIVE guidelines recommended for reporting animal research, and Sex and Gender in Research

Laboratory animals	NSG-SGM3 (NOD.Cg-Prkdcscid Il2rgtmWjlTg (CMV-IL3, CSF2, KITLG) 1Eav/MloySzJ, NSGS, The Jackson Laboratory) mice were purchased from Jackson Laboratory (The Jackson Laboratory)
Wild animals	N/A
Reporting on sex	The experimental groups within single experiment were sex-matched to reduce experimental variance (per common practice), but sex was not considered as a determining factor.
Field-collected samples	N/A
Ethics oversight	All experiments were conducted as previously described and in the accordance with the guidelines of the Regierung von Oberbayern. All protocols were approved by Institutional Animal Care and Use Committee of the Regierung von Oberbayern (ROB-55.2-2532.Vet_02-17-138 and Vet_02-18-162).

Note that full information on the approval of the study protocol must also be provided in the manuscript.

Flow Cytometry

Plots

Confirm that:

- [x] The axis labels state the marker and fluorochrome used (e.g. CD4-FITC).
- [x] The axis scales are clearly visible. Include numbers along axes only for bottom left plot of group (a 'group' is an analysis of identical markers).
- [x] All plots are contour plots with outliers or pseudocolor plots.
- [x] A numerical value for number of cells or percentage (with statistics) is provided.

Methodology

Sample preparation	Blood donations were source of cells of interest (T cells). T cells were isolated using in-house selection technology as previously described (Radisch et al., Sci Rep 2020). Pure T cell population was prepared according to standard antibody staining protocol. Briefly, cells were washed with PBS+0.5% BSA and incubated with antibody mix for 20 min at 4C. Afterward samples were washed twice and resuspended in the same buffer for acquisition.
Instrument	Cytoflex LX (Beckman Coulter)
Software	Flow cytometric data were analyzed using CytExpert (Beckman Coulter) and FlowJo software (FlowJo, LLC)
Cell population abundance	N/A

Gating strategy	Forward scatter (FSC) and side scatter (SSC) gate is set to exclude debris and non-cellular events. Then, a lymphocyte gate is set to exclude other cell types such as doublets, residual monocytes and granulocytes. Next, samples are gated on PI negative population to exclude dead cells. Finally, a gate is set on the CD45+CD3+ population to identify T cells. Further gating is performed depending on experimental requirements.

☐ Tick this box to confirm that a figure exemplifying the gating strategy is provided in the Supplementary Information.
